# MIS-NeRF: neural radiance fields in minimally-invasive surgery

**DOI:** 10.1007/s11548-025-03429-7

**Published:** 2025-05-25

**Authors:** Samad Barri Khojasteh, David Fuentes-Jimenez, Daniel Pizarro, Yamid Espinel, Adrien Bartoli

**Affiliations:** 1 Department of Electronics, Universidad de Alcala, Alcala de Henares, Madrid Spain; 2 DIA2M-DRCI, CHU, Clermont-Ferrand, France

**Keywords:** Intraoperative 3D reconstruction, 3D–2D registration, Liver, Laparoscopy

## Abstract

****Purpose**:**

Minimally-invasive surgery (MIS) reduces the trauma compared to open surgery but is challenging for endophytic lesion localisation. Augmented reality (AR) is a promising assistance, which superimposes a preoperative 3D lesion model onto the MIS images. It requires solving the difficult problem of 3D model to MIS image registration. We propose MIS-NeRF, a neural radiance field (NeRF) which provides high-fidelity intraoperative 3D reconstruction, used to bootstrap iterative closest point (ICP) registration.

****Methods**:**

Existing NeRF methods break down in MIS because of the moving light source and specular highlights. The proposed MIS-NeRF is adapted to these conditions. First, it incorporates the camera centre as an additional input to the radiance function, which allows MIS-NeRF to handle the moving light source. Second, it uses a modified volume rendering which handles specular highlights. Third, it uses a regularised compound loss to enhance surface reconstruction.

****Results**:**

MIS-NeRF was tested on three synthetic datasets and retrospectively on four laparoscopic surgeries. It successfully reconstructed high-fidelity liver and uterus surfaces, reducing common artefacts including high-frequency noise and bumps caused by specular highlights. ICP registration achieved faithful alignment between the preoperative and intraoperative 3D models, with an average error of 3.25 mm, outperforming the second-best method by a $15\%$ margin.

****Conclusion**:**

MIS-NeRF improves AR-based lesion localisation by facilitating accurate 3D model registration to multiple MIS images.

## Introduction

Minimally-invasive surgery (MIS) offers significant benefits over open surgery by reducing patient trauma and shortening length of stay. However, endophytic lesions, which are internal to an organ, are difficult to localise from the MIS images only due to reduced haptics [1–3]. Augmented reality (AR) superimposes the patient’s preoperative 3D model, reconstructed from MRI or CT scans, onto live MIS images, providing surgeons with valuable spatial context. This functionality depends on the ability to solve the 3D–2D registration problem, which involves aligning the preoperative 3D model to the MIS 2D images [4]. The required accuracy of a surgical AR system depends on the procedure. In hepatectomy, posterior liver tumours (e.g. hepatocellular carcinoma) cannot be localised by endoscopic ultrasound, so a 1 cm oncologic margin sets the AR accuracy requirement [5]. In myomectomy, small myomas under 1 cm are difficult to localise and may require a follow-up surgery if missed [6]. Thus, a registration accuracy of 1 cm is set for AR on the liver and the uterus.

Existing methods for 3D–2D registration in MIS have two main limitations. The first limitation is their reliance on identifying *anatomical landmarks* within the preoperative 3D model and the intraoperative images [1, 2, 7]. For the liver, typical landmarks include the falciform ligament and the inferior ridge. Detecting these landmarks in MIS images is challenging due to inter-patient variability and limited visibility; furthermore, it requires the annotation of dozens of points on each intraoperative image, demanding substantial domain expertise. Consequently, using only landmarks often fails to adequately constrain the registration, as this approach neglects other visual information crucial for accurate alignment. The second limitation is the use of a *single image* for registration. Early monocular methods [1, 2, 8] employed non-convex numerical optimisation to minimise the landmark reprojection error. However, these methods require manual initialisation and are prohibitively slow for surgery, as they necessitate optimisation and landmark annotation for every intraoperative frame. More recent deep learning methods [4, 9] reduce manual effort but can lack accuracy under realistic surgical conditions. Alternatively, stereo-image and multimonocular-image methods have been proposed [10, 11]. These approaches reconstruct the organ surface via structure-from-motion (SfM) and multiview stereo (MVS) [12]. They then solve a 3D–3D registration problem using the preoperative model, which is potentially more accurate than single-frame approaches [13]. Multiview strategies usually take a few minutes to perform the initial registration, after which robust real-time tracking can be used to update it. This workflow is clinically relevant for organs such as the uterus and the liver, where registration typically only needs to be performed once at the beginning of surgery. Nevertheless, these techniques often underperform in MIS, yielding very low-density SfM reconstructions. This is primarily due to the sparsity of inter-image point correspondences under challenging MIS imaging conditions, which include specularities, moving light sources, limited texture and difficult camera motion.

We address both limitations by performing registration without landmarks and by combining multiple images, whether monocular or stereo. To this end, we propose MIS-NeRF, a neural radiance field (NeRF) [14] framework specifically adapted to the challenging lighting and scene characteristics of MIS. This framework produces smooth and detailed reconstructions of the intraoperative 3D surface. Existing NeRF methods typically perform poorly in MIS due to varying brightness (caused by the moving light source) and significant specular reflections (from moist tissues). While an endoscopic adaptation of NeRF [15] and LightNeuS [16] show promise, they either rely on depth supervision or assume nearly watertight, cylindrical anatomy typical of colonoscopy; assumptions often unsuitable for general MIS applications.

The proposed MIS-NeRF is adapted to the MIS conditions via three main contributions. Firstly, the camera centre is incorporated as an input to account for the moving light source. Secondly, the camera response function is explicitly modelled during volume rendering. Thirdly, a compound loss function is introduced which excludes specular highlights from the colour-error term and incorporates depth smoothing to reduce surface reconstruction errors. Although [15] also incorporates the camera origin, it focuses on synthetic colonoscopy images and requires depth supervision. In contrast, MIS-NeRF targets surface reconstruction and trains directly from real images without depth priors.

We use the surfaces generated by MIS-NeRF to feed a 3D–3D registration method based on the iterative closest point (ICP) algorithm. This aligns the intraoperative and preoperative surfaces and requires minimal manual initialisation, contrasting with existing MIS registration methods that necessitate extensive point annotation and rely heavily on medical expertise. The complete MIS-NeRF and ICP method takes approximately 15 minutes to complete both reconstruction and registration, rendering it suitable for application within the surgical workflow in organs like the liver or the uterus. Furthermore, a key advantage is that the intraoperative model is reconstructed only once. Subsequently, additional intraoperative frames can be incorporated to track the organ without requiring further optimisation. The code and data for our methods will be made publicly available.

## Background

**Fig. 1:**
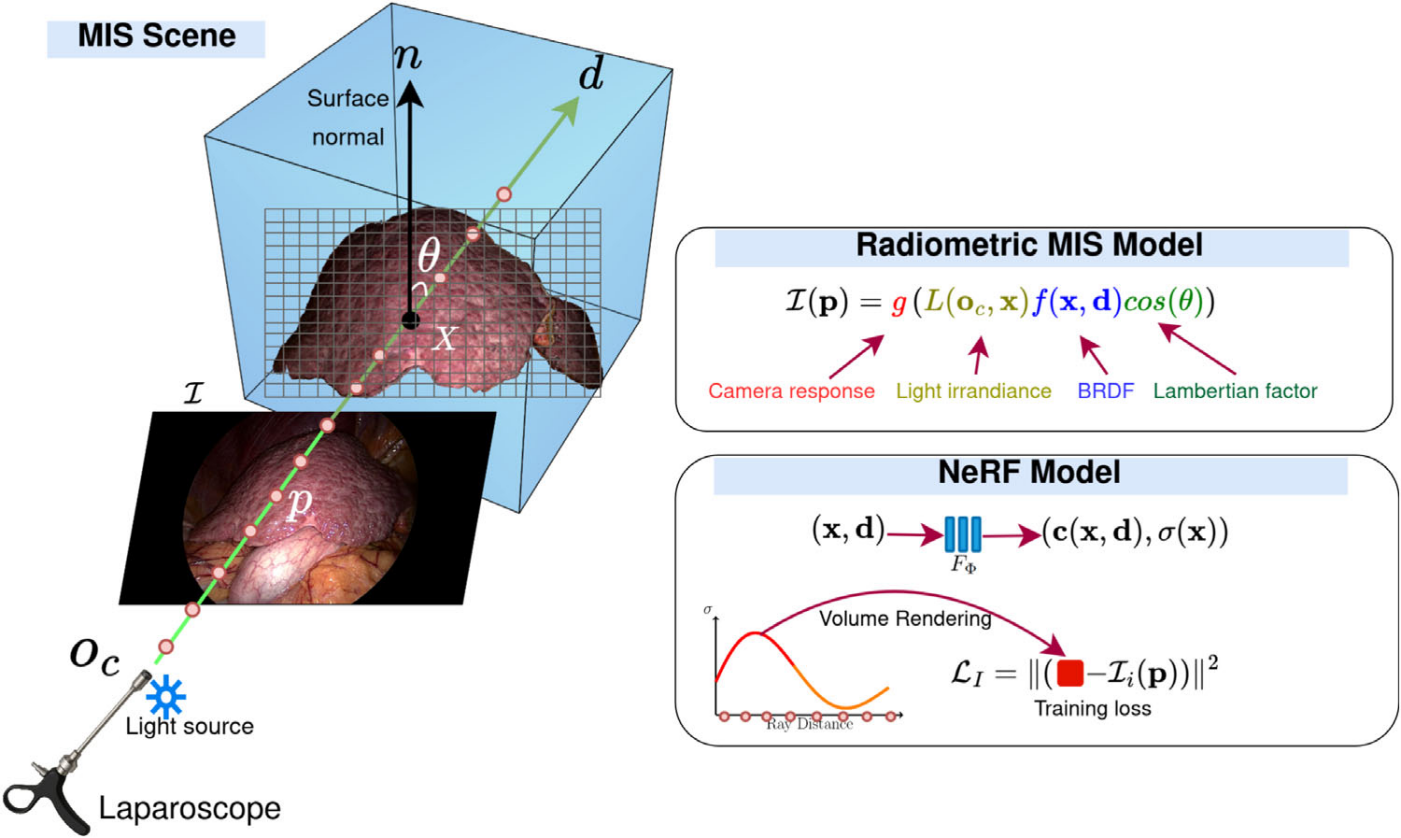
The MIS setup involves a laparoscope with embedded light source and the organ surface. The radiometric model includes the light’s irradiance, the surface BRDF and the camera response. The NeRF model uses volume rendering to represent pixel colour intensities along 3D rays. It is trained using direct colour discrepancy

We give background in the radiometric modelling of MIS and the NeRF principles. Fig. 1 shows a diagram with the main elements involved in this section.

### Radiometric MIS model

A specificity of MIS is that the light source is approximately collocated and moving with the camera. It can be reliably modelled as a spotlight positioned at the camera centre [11]. The image colour measured at pixel $\textbf{p}$, corresponding to surface point $\textbf{x}$, is:

1 $$\begin{aligned} \mathcal {I}(\textbf{p})=g\left( L(\textbf{o}_c,\textbf{x}) f(\textbf{x},\textbf{d}) \cos (\theta )\right) , \end{aligned}$$

where $\textbf{o}_c$ is the camera centre, *f* is the bidirectional reflectance distribution function (BRDF) and $\textbf{d}$ is the viewing direction. By positioning the spotlight at the camera centre, $\textbf{d}$ is also the direction of the incoming light ray at surface point $\textbf{x}$. The Lambertian factor $\cos (\theta )=-\textbf{d}\cdot \textbf{n}$ weights the irradiance at the surface given the surface normal $\textbf{n}$ and the light direction. The spotlight irradiance distribution *L* is:

2 $$\begin{aligned} L=\frac{L_e}{\mu ^\alpha } \quad \text {with} \quad \mu =\Vert \textbf{x}-\textbf{o}_c\Vert , \end{aligned}$$

where $L_e$ is the emitted radiance, $\mu$ the distance from the light source to the surface point and $\alpha$ the radiance decay factor. The camera response function *g* converts the received surface irradiance to RGB pixel colour. For simplicity, we use a model affecting all channels equally as:

3 $$\begin{aligned} g(\textbf{z})=\tanh \left( (g_0\textbf{z})^{\frac{1}{\gamma }}\right) , \end{aligned}$$

where $g_0$ is a constant camera gain factor, $\gamma$ is the gamma correction and $\tanh$ is a saturation function, as the irradiance is always positive. Consequently, the modelled camera response is nearly linear for low irradiance values and saturates for high irradiance values. We normalise the colour channel intensities within the interval [0, 1].

### Neural radiance fields

NeRF represents the scene with a 5*D* continuous vectorial function $(\textbf{c}(\textbf{x},\textbf{d}),\sigma (\textbf{x}))$, where $\textbf{c}=(c_R,c_G,c_B)$ is the radiance emitted in viewing direction $\textbf{d}(\theta ,\phi )$ at 3D point $\textbf{x}=(x, y, z)^\top$ and $\sigma (\textbf{x})$ can be interpreted as the probability of a ray terminating at an infinitesimal particle located at $\textbf{x}$. These functions are represented by a multilayer perceptron (MLP), $(\textbf{c},\mathbf {\sigma })=F_{\Phi }(\textbf{x},\textbf{d})$ with trainable parameters $\Phi$. NeRF implements volume rendering to predict the RGB colour components $\textbf{C}(r)=(C_R, C_G, C_B)^\top$ along a ray $\textbf{r}(t)=\textbf{o}+t \textbf{d}$, $t\in [t_n,t_f]$ with origin $\textbf{o}=(x_o,y_o,z_o)$ and direction $\textbf{d}$, as:

4 $$\begin{aligned} \textbf{C}(r)= &   \int _{t_n}^{t_f} T(t)\; \sigma (\textbf{r}(t)) \; \textbf{c}(\textbf{r}(t),\textbf{d})\;\textrm{d} t \quad \text {where} \nonumber \\ T(t)= &   \exp \left( -\int _{t_n}^{t} \sigma (\textbf{r}(\tau )) \textrm{d}\tau )\right) . \end{aligned}$$

The term *T*(*t*) acts as a density probability distribution along the ray. The surface depth $D(\textbf{r})$ can also be obtained by integration, as:

5 $$\begin{aligned} D(\textbf{r})= \int _{t_n}^{t_f} T(t)\; \sigma (\textbf{r}(t)) \;t\; \textrm{d}t. \end{aligned}$$

The MLP parameters $\Phi$ are ‘trained’ from multiple images $\mathcal {I}_i$ with $i=1,\dots ,N_f$. The training loss is the squared error between the rendered and observed pixel colours:

6 $$\begin{aligned} \mathcal {L}_I=\sum _{i=1}^{N_f}\sum _{\textbf{p}\in \Omega _i} \Vert \textbf{C}(\textbf{r}_i(\textbf{p})) - \mathcal {I}_i(\textbf{p})\Vert ^2, \end{aligned}$$

where $\textbf{r}_i(\textbf{p})$ is the ray corresponding to pixel coordinates $\textbf{p}$, sampled from the image region $\Omega _i$ in the *i*-th image. The ray is described in a 3D coordinate system common to all images, available from SfM.

**Fig. 2:**
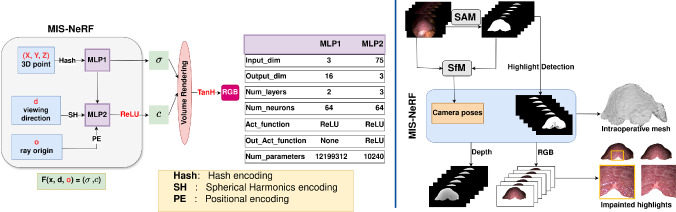
(left) The proposed MIS-NeRF architecture, which adds the camera centre as input, uses a ReLU activation for the colour density output, and $\tanh$ to model camera saturation after volume rendering. (right) MIS-NeRF training flowchart

## Materials and methods

We give an analysis of the modelling error incurred by using the existing NeRF implementations in the MIS context. We then propose the MIS-NeRF architecture to fix these errors using an appropriate model.

### Modelling-error analysis

The challenging MIS conditions often cause reconstruction errors, owing to the modelling approximations in the original NeRF formulation. We identify two challenges. **1) MIS radiometric modelling.** In NeRF, the radiance field is sampled along a ray to render the pixel colour, potentially learning elements of the radiometric model including the BRDF and the Lambertian factor. However, the dependency on the camera origin $\textbf{o}_c$ of the radiometric model, as shown in equation (1), cannot be learnt from the input parameters, leading to errors when attempting to fit the training RGB images. This propagates errors into the density distribution $\sigma$ and affects the geometry of the reconstructed scene. **2) High-frequency density errors**. NeRF reconstructions in MIS exhibit high-frequency surface errors despite accurate RGB rendering. We observed that this is due to three main factors. First, the large textureless areas in the organs, which create ambiguities in the learnt density distribution $\sigma$. Second, the moist shiny surfaces, which produce specular highlights where the surface normal $\textbf{n}$ and light ray $\textbf{d}$ align, resulting in white, saturated areas. While NeRF can reproduce highlights, the $\sigma$ distribution at these points is ill-defined, leading to bumps and holes in the surface. Third, the limited motion range of the MIS camera, as constrained by the organ and keyhole, creates a small baseline complicating the learning of scene geometry.

The proposed MIS-NeRF addresses both challenges using a radiance model with an explicit spotlight and saturated camera response, and a surface smoothing prior.

### MIS-NeRF architecture

MIS-NeRF introduces two architectural changes to NeRF: first, it adds $\textbf{o}_c$ as an input to the field network, and second, it replaces the sigmoid output layer with a ReLU function, modifying the volume rendering equation to account for camera saturation. MIS-NeRF introduces two changes to the NeRF loss function: first, it detects and removes specular highlights, and second, it incorporates depth smoothing into a compound loss. We describe these improvements directly below.

#### MIS-NeRF field function

The base architecture of MIS-NeRF is inspired by Nerfacto [17]. Following section 2.1, MIS-NeRF adds the camera origin $\textbf{o}_c$ as extra input to model light attenuation as:

7 $$\begin{aligned} \left( \textbf{c}(\textbf{x},\textbf{d},\textbf{o}_c \right) ,\sigma (\textbf{x}))=F_{\Phi } (\textbf{x},\textbf{d},\textbf{o}_c). \end{aligned}$$

Function $F_\Phi$ is represented by several encoders and two MLP blocks, as shown in Fig. 2. A hash encoding is used for the position $\textbf{x}$, followed by a first small-scale MLP to generate $\sigma$. A spherical harmonics encoding is used for the ray direction $\textbf{d}$ and a positional encoding is used for the camera centre $\textbf{o}_c$, which are both fed into a second MLP, along with the internal embedding from the first MLP, to produce the colour density output $\textbf{c}$.

We change the sigmoid output layer for the colour density $\textbf{c}$ to a ReLU, which clips the intensity from the bottom but allows it to overflow from the top. At this stage indeed, the physical irradiance must be positive, but should not be bounded to a particular energy upper-bound. Only at the end of rendering should the irradiance be upper-bounded, which physically occurs owing to the camera sensor saturating in over-exposed pixels. We explicitly model this saturation by adding the post-rendering $\tanh$ function.

**Fig. 3:**
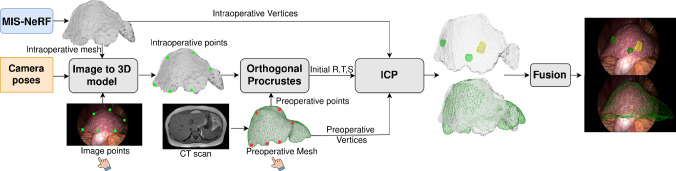
Proposed registration method using MIS-NeRF and ICP

#### Volume rendering with camera saturation

Following section 3.2.1, the proposed MIS-NeRF field function removes saturation from the field equation. For that, we change the existing sigmoid output activation, used in existing implementations, by the unbounded ReLU function. We then explicitly model and incorporate the tanh saturation model described in equation 3, to the rendering equation as:

8 $$\begin{aligned} \textbf{C}(r)=\tanh \left( \int _{t_n}^{t_f} T(t)\; \sigma (\textbf{r}(t)) \; \textbf{c}(\textbf{r}(t),\textbf{d},\textbf{o}_c)\; \textrm{d}t \right) . \end{aligned}$$

These modifications allow the camera gain and gamma correction to be learnt by the neural network representing $\textbf{c}$. They thus allow the colour and density distributions to produce high-intensity colours for each ray without explicitly handling saturation. Experimental results reveal that these modifications improve the results of the original NeRF architecture in MIS conditions, enabling the system to better learn and reconstruct the structure of the scene around saturated areas. This is for instance clearly visible in the reconstructed images shown in Figs. 5 and 6.

#### Training loss

We train MIS-NeRF using a compound loss $\mathcal {L}_T = \mathcal {L}_I + \lambda \mathcal {L}_s$. The colour-error term $\mathcal {L}_I$ is defined in equation (6) and the smoothing term $\mathcal {L}_s$ is:

9 $$\begin{aligned} \mathcal {L}_s= &   \sum ^{N_f}_{i=1}\sum _{\textbf{p}\in \Omega _i} \left( |D(\textbf{r}_i(\textbf{p}))-D(\textbf{r}_i(\textbf{p}+\mathbf {\Delta _x}))|\right. \nonumber \\  &   \left. +|D(\textbf{r}_i(\textbf{p}))-D(\textbf{r}_i(\textbf{p}+\mathbf {\Delta _y}))| \right) , \end{aligned}$$

where $\textbf{r}_i(\textbf{p})$ is the ray corresponding to pixel $\textbf{p}$ in the *i*-th camera, and $D(\textbf{r})$ represents the depth synthesised by the MIS-NeRF MLP. The pixel displacements $\mathbf {\Delta _x}$ and $\mathbf {\Delta _y}$ represent the 4-neighbour pixel rays.

In MIS-NeRF, saturated highlight areas are removed from the region $\Omega _i$, improving surface reconstruction and enabling automatic highlight inpainting when synthesising new views. MIS-NeRF fills in these areas using information from the other images where the area shows diffuse reflection. In our experiments, we used the segment anything model (SAM) [18] for foreground masking and segmented the highlights with simple intensity thresholding.

**Fig. 4:**
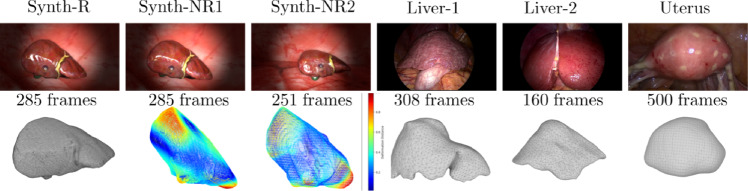
(top) MIS images and number of frames in each dataset. (bottom) preoperative 3D models with deformation heat maps shown for Synth-NR1 and Synth-NR2

## Using MIS-NeRF for registration

We describe the proposed method to register the preoperative 3D organ model to the MIS images, shown in Fig. 3. Recall that the preoperative 3D model is a 3D surface mesh. We first run SfM on the surgical images, which provides camera pose and intrinsic parameters. We then fit MIS-NeRF to the surgical images using the method from section 3.2. Once trained, we extract the intraoperative 3D surface from the MIS-NeRF field function by following [17]. This involves uniformly sampling a point cloud from the MIS-NeRF density function within a volume covering the scene, estimating point-cloud normals from neighbouring points, and applying Poisson surface reconstruction to produce a 3D triangular mesh of the intraoperative surface. Lastly, we use ICP to find the similarity transformation, composed of scale *s*, rotation $R\in SO(3)$ and translation $T\in \mathbb {R}^3$ between the preoperative and intraoperative 3D models. ICP requires a coarse initialisation, which we provide using Procrustes analysis [19] from a small set of at least 4 manually selected point correspondences, with one-point set specified on the preoperative 3D model and one-point set specified on any MIS image and automatically reconstructed using the intraoperative 3D model. This process does not need to be very accurate since coarsely aligning the models is enough to bootstrap ICP. Given the registration, the preoperative model is transferred and projected onto the MIS images to realise AR.

## Experimental results

We present the datasets, the evaluation metrics and compared methods, and report the results.

### Datasets

We use three synthetic liver MIS datasets and four real MIS datasets. Synthetic images are rendered in Blender with a realistic 3D liver model. The laparoscope is modelled as a pin-hole camera with lens distortion and a co-moving spotlight, replicating real constraints. The chosen BRDF produces specular reflections. We follow three deformation settings: Synth-R (rigid), Synth-NR1 (torsion forces, average deformation of $6.8 \pm 3.3$ mm, maximum vertex displacement of 17.8 mm) and Synth-NR2 (forces applied by instruments average deformation of $7.4 \pm 3.9$ mm, maximum vertex displacement of 21.8 mm). Liver-1, Liver-2, Liver-3 and Uterus are videos extracted from four MIS procedures^1^, showing deformations induced by breathing, neighbouring organs and motion of the uterus caused by an external cannula. Preoperative 3D models of the liver and uterus were reconstructed from MRI data using MITK. We uniformly sample frames from each MIS video, shown in Fig. 4, leaving $10\%$ of these images for testing, with the remainder used for training. Liver-3 is shown in Fig. 6 as a failure case.

### Evaluation metrics and methods

We use five metrics. PSNR, SSIM and LPIPS are standard image reconstruction metrics [14]. PSNR measures pixel-level colour fidelity on a logarithmic scale, SSIM captures structural similarities in luminance and contrast and LPIPS uses deep features to quantify perceptual differences. *High PSNR and SSIM and low LPIPS indicate good rendering quality.* The other two metrics are the ICP registration error and the surface depth error. The ICP error is computed one-sided, as the intraoperative 3D model only covers the visible portion of the organ. It is defined as the average distance between each vertex on the intraoperative surface and its corresponding point on the registered preoperative surface. The depth RMSE is the discrepancy between the reconstructed and the ground-truth surfaces:

10 $$\begin{aligned} \textrm{RMSE}({\bar{D}})=\sqrt{\frac{1}{m}\sum _{\textbf{p}\in \Omega } \left( D(\textbf{r}(\textbf{p})) - \bar{\alpha } \bar{D}(\textbf{r}(\textbf{p})) \right) ^2 }, \end{aligned}$$

where $\Omega$ is the liver image region, *D* and $\bar{D}$ are the ground-truth and estimated depth values and $\bar{\alpha }$ adjusts for scale differences between the reconstruction and ground-truth. The depth RMSE requires the ground-truth surface and is thus available only on synthetic dataset experiments. *Low ICP error and depth RMSE indicate good registration quality.*

We compare the original Nerfacto [17] with several variants. These are constructed from the options Act (changes to ReLU for colour density and volume rendering with saturation), Origin (adds camera origin to the field function), Smoo (adds the smoothing loss term) and Spe (removes specular regions from ray sampling). Combinations of these options are noted as X+Y or X+Y+Z, where X, Y and Z are chosen from {Act, Origin, Smoo, Spe}. MIS-NeRF corresponds to Spe+Smoo+Act+Origin. Comparing all these variants is thus an ablation study of MIS-NeRF. We also compare with two state-of-the-art surface reconstruction methods: MVS [12] followed by Poisson surface reconstruction, previously used in MIS registration, and SuGaR [20], a recent method based on 3D Gaussian Splatting.

### Results in synthetic and real datasets

**Fig. 5:**
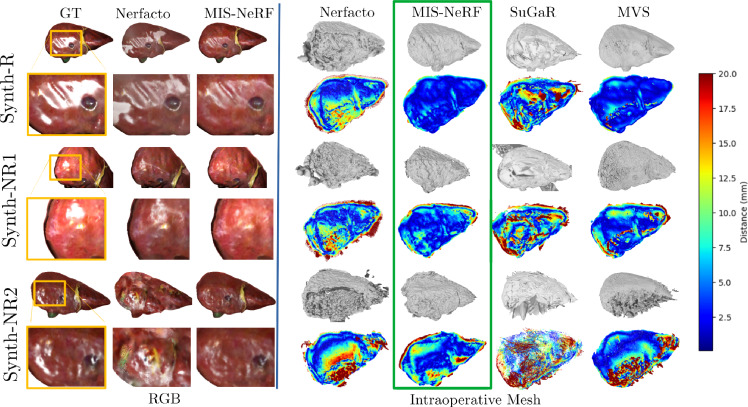
Results in the synthetic datasets. (left) RGB synthesis of a test image with close-up on a highlight-contaminated region. (right) Reconstructed shaded intraoperative 3D models and ICP error heat map capped at 20 mm

Table 1 shows results on the synthetic datasets. MIS-NeRF consistently outperforms Nerfacto and the other variants (X+Y+Z) across all metrics in Synth-R, demonstrating superior image fidelity, perceptual quality and surface reconstruction accuracy. Spe+Origin is close to MIS-NeRF in terms of image fidelity but exhibits higher depth RMSE and ICP errors, highlighting the importance of the activation function (Act) and Smoothing (Smoo) innovations. MIS-NeRF also outperforms SuGaR and Nerfacto in depth RMSE and ICP errors across all three synthetic datasets. The results for MIS-NeRF and MVS are comparable. On average for the three synthetic data experiments (Synth-R, Synth-NR1 and Synth-NR2), MVS achieves a slightly better depth RMSE (14.89 mm versus 16.28 mm for MIS-NeRF) but a significantly worse ICP error (4.77 mm versus 3.81 mm for MIS-NeRF). This trend is consistent in Synth-R and Synth-NR2; however, in Synth-NR1, the trend is reversed, with MIS-NeRF exhibiting a slightly lower depth RMSE with a marginally greater ICP error. Fig.5 shows that MIS-NeRF produces smoother surfaces and robust highlight inpainting in the synthesised test images.

**Table 1:** Quantitative results for synthetic data. Bold is best

Methods	PSNR$\uparrow$	SSIM$\uparrow$	LPIPS$\downarrow$	Depth RMSE$\downarrow$ (mm)	ICP Error$\downarrow$ (mm)
**Synth-R**					
Nerfacto	29.40	0.989	0.008	23.07	8.39
Act	29.51	0.986	0.010	17.45	7.32
Origin	32.75	0.993	0.005	22.33	8.17
Smoo	29.39	0.989	0.008	21.62	8.08
Spe	29.75	0.994	0.004	16.18	2.71
Smoo+Act	29.57	0.986	0.010	17.51	6.25
Smoo+Origin	33.13	0.993	0.005	21.26	7.00
Spe+Act	30.61	0.993	0.004	13.61	2.33
Spe+Origin	33.27	$\mathbf {0.996}$	0.002	16.29	2.52
Spe+Smoo	29.68	0.993	0.005	14.53	2.51
Spe+Smoo+Act	30.18	0.992	0.004	13.69	2.58
Spe+Smoo+Origin	33.65	$\mathbf {0.996}$	$\mathbf {0.002}$	15.16	2.44
**MIS-NeRF**	$\mathbf {34.02}$	$\mathbf {0.996}$	$\mathbf {0.002}$	13.19	$\mathbf {2.27}$
SuGaR	−	−	−	14.78	4.93
MVS	−	−	−	$\mathbf {11.66}$	2.71
**Synth-NR1**					
Nerfacto	28.23	0.979	0.014	24.69	10.10
**MIS-NeRF**	$\mathbf {31.51}$	$\mathbf {0.989}$	$\mathbf {0.006}$	$\mathbf {15.97}$	4.16
SuGaR	−	−	−	18.78	6.20
MVS	−	−	−	16.31	$\mathbf {3.83}$
**Synth-NR2**					
Nerfacto	24.71	0.992	0.004	21.83	10.21
**MIS-NeRF**	$\mathbf {35.46}$	$\mathbf {0.998}$	$\mathbf {0.001}$	19.70	$\mathbf {5.01}$
SuGaR	−	−	−	20.47	12.00
MVS	−	−	−	$\mathbf {16.69}$	7.79

**Table 2:** Quantitative results for the real datasets. Bold is best

**Liver**								
Methods	PSNR$\uparrow$		SSIM$\uparrow$		LPIPS$\downarrow$		ICP Error$\downarrow$ (mm)	
	Liver-1	Liver-2	Liver-1	Liver-2	Liver-1	Liver-2	Liver-1	Liver-2
Nerfacto	29.33	30.86	0.970	0.933	0.022	0.048	3.73	4.80
Act	29.86	31.86	0.972	0.937	0.021	0.047	3.16	6.45
Origin	34.16	34.63	0.981	0.963	$\mathbf {0.013}$	0.026	2.95	3.94
Smoo	29.28	29.42	0.969	0.928	0.023	0.051	2.62	5.18
Spe	29.27	30.00	0.969	0.937	0.022	0.045	4.27	5.25
Smoo+Act	29.86	31.83	0.973	0.939	0.021	0.047	2.68	3.94
Smoo+Origin	34.28	34.56	0.982	0.963	0.014	0.026	2.63	3.78
Spe+Act	29.66	32.15	0.973	0.937	0.020	0.048	3.37	4.74
Spe+Origin	34.59	35.07	$\mathbf {0.983}$	0.967	$\mathbf {0.013}$	0.024	3.58	4.31
Spe+Smoo	29.41	29.05	0.969	0.928	0.022	0.049	2.56	4.63
Spe+Smoo+Act	29.68	32.10	0.973	0.940	0.021	0.044	3.30	3.62
Spe+Smoo+Origin	34.58	35.59	0.982	$\mathbf {0.967}$	0.014	$\mathbf {0.023}$	2.55	3.73
**MIS-NeRF**	$\mathbf {34.82}$	$\mathbf {35.73}$	$\mathbf {0.983}$	$\mathbf {0.967}$	$\mathbf {0.013}$	$\mathbf {0.023}$	$\mathbf {2.48}$	3.66
SuGaR	−	−	−	−	−	−	2.73	4.62
MVS	−	−	−	−	−	−	2.59	$\mathbf {3.49}$

**Uterus**								
Methods	PSNR$\uparrow$		SSIM$\uparrow$		LPIPS$\downarrow$		ICP Error$\downarrow$ (mm)	
Nerfacto	26.39		0.957		0.068		2.14	
**MIS-NeRF**	$\mathbf {33.48}$		$\mathbf {0.967}$		$\mathbf {0.067}$		$\mathbf {1.77}$	
SuGaR	−		−		−		2.66	
MVS	−		−		−		1.88	

**Fig. 6:**
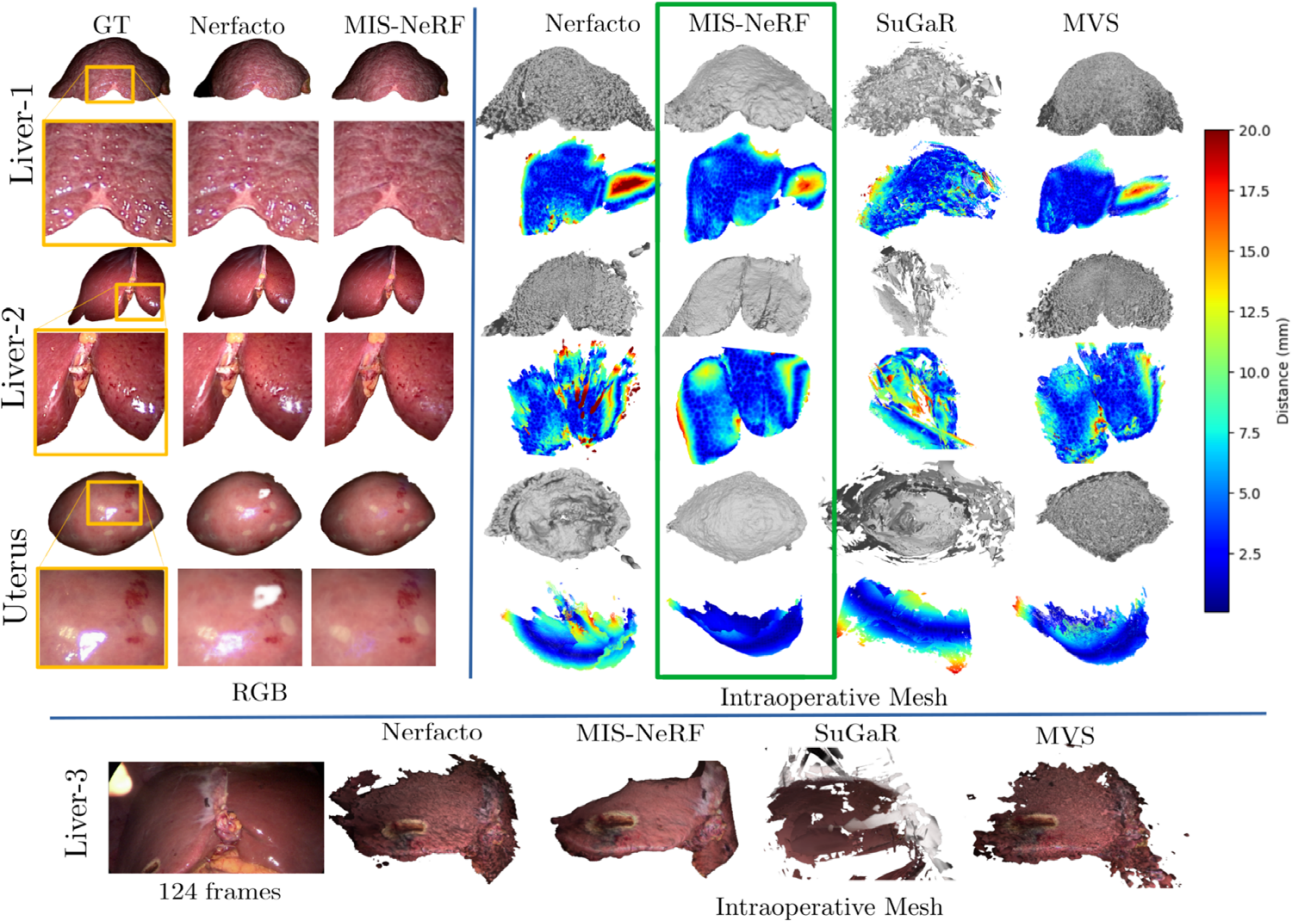
Results in the real datasets. (left) RGB synthesis of a test image with close-up on a highlight-contaminated region. (right) Reconstructed shaded intraoperative 3D models and ICP error heat map capped at 20 mm. The bottom row shows a failure case in the Liver-3 dataset

Table 2 shows results on the real datasets. The findings align with the synthetic data: MIS-NeRF outperforms the other NeRF variants across all image quality and ICP metrics, confirming the contributions of the proposed modules. Compared to SuGaR and MVS, MIS-NeRF achieves the best ICP performance in Liver-1 and Uterus, and the second-best by 0.17 mm in Liver-2 compared to MVS. Fig. 6 shows qualitative examples, showing how MIS-NeRF effectively inpaints highlights and produces smoother surfaces than the other methods. In contrast, SuGaR produces noisier surfaces, likely due to the small baseline in the real datasets leading to sparse splatting. Fig. 6 also shows the registration error distribution, confirming consistently lower errors for MIS-NeRF, in line with table 2. Lastly, fig. 6 illustrates a failure case occurring in the Liver-3 dataset, where the camera motion is so reduced that all methods fail to recover the correct surface.

The average training times in all datasets are: Nerfacto, 12 mins; MIS-NeRF, 15 mins; SuGaR, 70 mins; and MVS, 45 mins. The ICP registration step, common to all methods, takes seconds. We used a hardware with a high-end Ryzen CPU and an Nvidia RTX3090 GPU. MIS-NeRF could be speeded up using recent strategies such as Instant-NGP [17]. There are also faster implementations of MVS. MIS-NeRF introduces an 11% increase in trainable parameters (10,240 parameters compared to Nerfacto’s 9,216), which does not affect training convergence.

## Conclusion

We have analysed the critical limitations to using the NeRF method in MIS and have addressed them to propose MIS-NeRF. MIS-NeRF handles a moving light source, specular highlights and camera saturation, obtaining precise intraoperative 3D surface reconstruction in MIS. Combined with ICP, it allows one to precisely register the preoperative 3D model. The proposed method does not use landmarks, a major limitation in previous work, and leverages the use of multiple MIS images. Experiments show that MIS-NeRF outperforms existing NeRF methods in both image fidelity and registration error, making it a promising tool for AR-based lesion localisation in MIS. Future work will automate registration by using a predefined initialisation pose, will replace SfM by SLAM, will speed up MIS-NeRF training and will leverage the high-fidelity image rendering capability of MIS-NeRF to propose live intraoperative virtual navigation for remote surgical assistance and surgeon education.

## Footnotes

1 Ethical approval IRB00008526-2019-CE58, CPP Sud-Est VI, Clermont-Ferrand, France.

## Declarations

### Code and datasets

The source code and materials used in this study are publicly available at https://github.com/samadbarrikhojasteh/MIS-NeRF.
